# Health Related Quality of Life amongst Sewerage and Sanitary Workers of Karachi, Pakistan

**DOI:** 10.12669/pjms.38.7.5697

**Published:** 2022

**Authors:** Syed Ishtiaq Ahmed Fatmi, Naveed Mansoori, Syed Muhammad Mubeen

**Affiliations:** 1 Dr. Syed Ishtiaq Ahmed Fatmi, MBBS, MPH. Department of Community Health Sciences, United Medical and Dental College, Karachi, Pakistan; 2 Dr. Naveed Mansoori, MBBS, MPH, MSc Biostatistics & Epidemiology. Department of Community Health Sciences, Hamdard College of Medicine & Dentistry, Hamdard University, Karachi, Pakistan; 3 Dr. Syed Muhammad Mubeen, MBBS, MES, MCPS, MPH. Department of Community Health Sciences, Hamdard College of Medicine & Dentistry, Hamdard University, Karachi, Pakistan

**Keywords:** Environment, Public health, Sanitary workers, Quality of life

## Abstract

**Objectives:**

To find out the impact of occupational and socio-demographic factors on the health related quality of life of sewerage and sanitary workers in Karachi.

**Methods:**

A cross-sectional study was conducted during 2019. Four hundred workers were chosen from five districts of Karachi using a non-probability convenient sampling technique. An Urdu version of WHO BRIEF quality of life questionnaire was used to collect the data about workers general health status and quality of life. Descriptive analysis was done and Chi-square test was used for the association of socio-demographic factors and quality of life. Multiple regression model was used to predict QOL of all domains. P-value<0.05 was considered as statistically significant.

**Results:**

Out of 400 sanitary workers, 228 (57.0%) were sweepers and the rest 172 (43.0) were sewerage workers. The majority of the workers 321 (80.3%) were male and 246 (61.5%) were illiterate. The vast majority of the workers 386 (96.5%) were not immunized against typhoid, / hepatitis and tetanus. Ninety-one percent (91%) were not using any kind of safety gadgets while at work. Male workers, married workers of both sexes and those with some education had a little better quality of life than their counterparts. Sanitary employees likewise had a higher quality of life score than sewage workers (P-value<0.05).

**Conclusion:**

The majority of workers, particularly sewage workers, have a very poor quality of life. Along with very bad working circumstances, their standard of living is significantly below par. They were not given any safety equipment. They were handling untreated sewage/waste with their bare hands, and they have never received a typhoid/hepatitis /tetanus vaccine.

## INTRODUCTION

World Health Organization (WHO) defines Quality of Life as individual’s perception of their place in life in relation to their goals, expectations, standards and concerns in the context of the culture and value systems in which they live.^1^ Since the 1980s, the concept of health-related quality of life (HRQOL) and its determinants has evolved to include those aspects of overall quality of life that can be clearly shown to affect health, which include physical, mental, and social domains and their correlates such as health risks and conditions, functional status, social support, and socioeconomic status.^2^

The occupation of an individual affects the individual’s wellbeing and, consequently, quality of life. Occupational factors such as job satisfaction, work environment, job stress, salary, working hours, working conditions, and job nature all have an impact on the individual.^3^ As of difference in work type and working conditions, health-related quality of life differs between occupational categories.^4^

Sanitary workers are the backbone of any society’s municipal cleaning system.^5^ Sweepers are workers who clean the roads and streets, while sewerage workers are those who maintain and clean the sewerage system. The cleaning process is highly mechanised in developed countries; but, in developing countries such as India and Pakistan, the cleaning technique remains human, even in urban vicinities with little resources.^6^ Because most workers in such localities lack modern sewage cleaning equipment, sewage workers enter the underground sewerage pipes through manholes and clear them whenever the lines become clogged for whatever reason. They enter the manholes almost naked, using a spliced bamboo pole (called *khapchi* in native language).^7^ In India every year, hundreds of men die as a result of harmful gases and lack of oxygen after inhaling toxic fumes from sludge flushed by millions of household, industries and offices in the metropolitan.^8^,^9^

Sweepers are exposed to a lot of debris, all forms of waste materials such as dirt, infective organisms, and other hazardous elements such as chemicals, animal excreta, and sharp objects while cleaning streets, producing injuries with serious repercussions.^10^ These workers are affected with a variety of chronic health issues. Respiratory symptoms, irritation of the skin, nose and eyes, gastrointestinal problems, various types of dermatitis, fatigue, chronic headaches, musculoskeletal and psychological problems are all common within this working group.^11^,^12^

In Pakistan, both sewage and sanitary employees do their occupations manually, without the need of safety equipment. In Karachi, it is a normal practice for workers to enter the main hole bare-handed, even without gloves or face mask; to clear the clogged sewage lines. They are the city’s lowest-paid and most discriminated employees, and their homes and neighborhoods reflect this. Despite being exposed to work dangers and disease-causing bacteria, the majority of these daily wagers do not have access to medical facilities.^13^

The issues influencing the wellbeing and hence health related quality of life of these workers are poor living conditions, a poor work environment, low earnings, a lack of preventive measures at work, chronic diseases, and social discrimination by society. Therefore, the objective of this study was to investigate effects of occupational and socio-demographic factors on the health-related quality of life (QOL) of sanitary workers of Karachi.

## METHODS

This cross sectional study was carried out in all five districts (Central, East, West, South, & Malir) of Karachi during 2019. With 95% confidence interval, 5% margin of error and 50% anticipated prevalence, the sample size was found to be 384. However, keeping in view of rejection for participation and non-response rate, the sample size was increased by 5%. A total of 400 sanitary and sewerage workers aged 18 years and above who agreed to participate and were able to understand and communicate were included using non-probability convenient sampling technique. The data was gathered using a pre-tested, Urdu version of the WHOQOL-BREF (World Health Organization Quality of Life) questionnaire^14^ provided by WHO. The WHOQOL-BREF instrument is made up of 26 items, two of which assess overall quality of life and general health. The remaining 24 questions are classified as into four domains; physical, psychological, social relationships, and environmental. Each item is graded on a scale from one to five. The scores are then converted into a linear scale ranging from zero till hundred (0-100), with zero representing the least favorable quality of life and 100 being the most favorable.

The study was approved by Hamdard University’s Ethical Review Committee (HCM&D/CHS/190/2019), and a list of workers was provided by municipal offices in each of the Karachi’s five districts. One off-duty sanitary worker accompanied the researchers to the locations where these sanitary workers lived, especially to their union councils and workplaces. Several methods for information gathering were used such as conversations, observations, attending of a typical day at work in the area with the workers, spending time walking around the area and talking with the workers about what their daily routines consists of or visiting their residents for data collection also during their quality time on a weekend or holiday. Only workers who volunteered to take part in the study were included. Workers who were suffering from serious disease were excluded.

After ensuring that all forms were filled out completely, the data were entered in SPSS version 22. Descriptive statistics was used for calculating frequencies and percentages of related variables. The Chi-square test was used to determine the association of socio-demographic variables and quality of life. A multiple regression model was used to predict all domains of sewerage workers’ quality of life scores.

## RESULTS

Out of 400 participants, 149 (46.4%) were sweepers and the remaining 172 (53.6%) were sewerage employees. The majority of the workers 257 (64%) were under the age of 35 and 246 (61.5%) were illiterate; illiteracy was defined as the inability to read or write. Two hundred and forty nine (62%) worked at least eight hours per day. The majority of employees 326 (81.5%) were married. Ninety-six percent (96%) had not been immunized against typhoid, tetanus and hepatitis while the remaining were unsure whether they had been immunized or not. (Table-I) In general, the majority of workers have a very low quality of life in all domains. Male workers, married workers of both sexes and those with some education had a little better quality of life than their counterparts. Sweepers also had a higher life quality score than sewage workers (P-value <0.05).

**Table-I:** Demographic characteristics of study population by sex.

Variables	Male n (%) = 321	Female n (%) = 79	Total n (%) = 400	p-value*
***Age in Years***				
<25	52 (16.2)	3 (3.8)	55 (13.8)	
26 – 35	156 (48.6)	45 (57.0)	201 (50.2)	0.028
36 – 45	107 (33.3)	28 (35.4)	135 (33.8)	
>45	6 (1.9)	3 (3.8)	9 (2.2)	
***Marital Status***				
Married	262 (81.6)	64 (81.0)	326 (81.5)	
Unmarried	57 (17.8)	7 (8.9)	64 (16.0)	<0.001
Divorced/Widowed	2 (0.6)	8 (10.1)	10 (2.5)	
***Education level***				
Illiterate	188 (58.6)	58 (73.4)	246 (61.5)	
Primary	84 (26.2)	14 (17.7)	98 (24.5)	0.101
Middle	37 (11.5)	6 (7.6)	43 (10.8)	
Matric	12 (3.7)	1 (1.3)	13 (3.2)	
***Type of Work***				
Sweeper	149 (46.4)	79 (100.0)	228 (57.0)	<0.001
Sewerage	172 (53.6)	0 (0.0)	172 (43.0)	
***Working Hours/day***				
≤ 8	199 (62.0)	50 (63.3)	249 (62.2)	0.897
> 8	122 (38.0)	29 (36.7)	151 (37.8)	
***Monthly Income***				
≤ 15,000	157 (48.9)	44 (55.7)	201 (50.2)	0.316
> 15,000	164 (51.1)	35 (44.3)	199 (49.8)	
***Vaccination***				
Yes	12 (3.7)	2 (2.5)	14 (3.5)	0.999
No	309 (96.3)	77 (97.5)	386 (96.5)	
***Relative died during work in sewerage line***				
Yes	84 (86.2)	8 (10.1)	92 (23.0)	0.002
No	237 (73.8)	71 (89.9)	308 (77.0)	

* Chi-square as the test of significance

The majority of the 312 (78%) workers used an addictive substance, with smoking accounting for 44.8% and tobacco chewing accounting for 29.5%. Half of the 200 (50%) workers had one or more physical disease. Hypertension 15%, muscle and joint pain 11%, respiratory problems 10% and diabetes mellitus 7%. A huge number of workers 371 (93%), did not use any safety gadgets while at work. (Table-II)

**Table-II:** Association of addiction status, disease history and safety gadgets use by both gender.

Variables	Male n(%)=321	Female n(%)=79	Total n(%)=400
***Addiction***			
Smoking	173 (53.9)	6 (7.6)	179 (44.8)
Tobacco chewing	98 (30.5)	20 (25.3)	118 (29.5)
Naswar	7 (2.2)	0 (0.0)	7 (1.8)
Alcohol	2 (0.6)	0 (0.0)	2 (0.5)
Other	5 (1.6)	1 (1.3)	6 (1.5)
No addiction	36 (11.2)	52 (65.8)	88 (22.0)
***History of Disease*** Diabetes Mellitus	17 (5.3)	11 (13.9)	28 (7.0)
Hypertension	41 (12.8)	19 (24.1)	60 (15.0)
Respiratory problem	32 (10.0)	7 (8.9)	39 (9.8)
Migraine	5 (1.6)	4 (5.1)	9 (2.3)
Tuberculosis	7 (2.2)	0 (0.0)	7 (1.8)
Liver problem	12 (3.7)	1 (1.3)	13 (3.3)
Muscle/Joint pain	30 (9.3)	14 (17.7)	44 (11.0)
No Disease	177 (55.1)	23 (29.1)	200 (50.0)
***Use of safety gadgets***			
Gloves	9 (2.8)	4 (5.1)	13 (3.2)
Face mask	7 (2.2)	0 (0.0)	7 (1.8)
Long boots	5 (1.6)	0 (0.0)	5 (1.2)
Oxygen kit	4 (1.2)	0 (0.0)	4 (1.0)
Never used	296 (92.2)	75 (94.9)	371 (93.0)

The outcomes of multiple regression analysis used to predict QoL is shown in Table-III. Overall and general health QoL was slightly higher among literate than the illiterate workers (P<0.05). Workers aged 26 years and up showed higher environmental QoL. Males scored higher in the physical and social domain of quality of life (P <0.05). Physical and environmental QoL were higher for married workers of both sexes. Workers with greater wages have higher environmental QOL than workers with lower wages. However, working hours per day, vaccination status, and the death of co-workers on the job had no effect on QoL.

**Table-III:** Multiple regression model to predict QOL of domains of QOL scores of the participants.

Characteristics	Overall and General Health QOL		Physical Health		Psychological Health		Social Relationship		Environmental Health	

	(B)*	p-value	(B)*	p-value	(B)*	p-value	(B)*	p-value	(B)*	p-value
Age	0.038	0.893	-0.385	0.055	0.038	0.843	0.176	0.420	0.700	0.002
Gender	- 0.543	0.275	-0.814	0.021	0.184	0.587	-0.776	0.043	-0.506	0.204
Marital Status	0.429	0.304	0.684	0.020	0.538	0.059	0.444	0.166	0.943	0.005
Education level	0.745	0.001	0.207	0.185	0.317	0.036	0.428	0.012	0.677	0.000
Type of Work	-0.599	0.166	0.327	0.283	0.293	0.320	0.199	0.549	0.788	0.023
Working Hours/day	0.133	0.713	0.041	0.871	-0.438	0.076	-0.315	0.258	-0.788	0.007
Monthly Income	-0.291	0.464	0.375	0.179	0.249	0.358	0.015	0.961	0.948	0.003
Vaccination	-1.196	0.210	0.163	0.807	0.852	0.190	0.972	0.184	-0.553	0.468
Death of co-workers in sewerage lines	-0.069	0.882	0.147	0.654	0.281	0.376	-0.370	0.300	0.311	0.403
(Constant)	14.296	<0.001	12.645	<0.001	10.331	<0.001	12.923	<0.001	9.757	<0.001

* Regression coefficient.

## DISCUSSION

The purpose of this study was to assess the health-related quality of their life (HRQOL) of sanitary workers in Karachi and its relationship with occupational and socio-demographic characteristics. Interestingly, we observed that education, male gender and advance age of workers have better QOL. High prevalence of addiction, chronic diseases, and lack of use of personnel protective equipment’s associated with limitations in health related quality of life in sewerage and sanitary workers.

In this study, we discovered that male workers had higher QOL than female workers. This contradicts many studies in the general population, which found that non-working females had a higher quality of life than working females.^15^ Males have more freedom of movement in our male-dominated society; they frequently gather with their friends and co-workers in tea houses, restaurants, parks and so one, whereas female workers have no time for themselves because, in addition to working, they are also responsible for household work and rousing the children.

Sixty-one per cent (61%) of our study population was illiterate (unable to read or write), which is comparable to the Minority Rights Commission Report 2007, which reported that 82 % of sweepers in Pakistan are uneducated. Literacy is positively connected with quality of life in our study as well as several other studies.^16^,^17^ Concerning vaccination, a significant number of workers 386 (96.5%) are not vaccinated for endemic diseases. This result is similar with Hamid MA,^18^ in Qalyobia who reported that none of the 140 workers are vaccinated (100%) for any prevalent diseases.

In our study, more than two-third of the workers were addicted to smoking, tobacco chewing with beetle nut, or niswar. These workers had a lower quality of life than non-addicts. This is congruent with findings in the general population, where several studies have found an inverse association between addiction and quality of life.^19^,^20^ There are various factors that contribute to the link between smoking and poor quality of life. These existing low-wage workers spent a considerable percentage of their pay on cigarettes, leaving less money to live in a country with rapid inflation and high prices for daily necessities. They also have a number of psychological issues that limit their capacity to work.

Sewerage workers also had adverse morbidity profile. The most common non-communicable diseases are hypertension (15%), musculoskeletal problems (11%), respiratory problems (9.8%) and diabetes mellitus (7.0%). Previous studies have also reported the high prevalence of chronic diseases.^21^,^22^ This might be explained through high work-related stress, exposure against toxins and allergens and addiction.

As regards the use of safety devices, the current study found that the majority of workers 371 (93%) were not wearing any personal protective equipment such as long boots, gloves, gowns, helmets, or masks while performing their duties of sweeping the street and opening clogged sewage system drains. Similar conditions exist in our neighboring country India, where researchers discovered that sanitation personnel were not wearing any safety equipment.^18^,^23^,^24^ The key variables influencing its implementation are lack of awareness, a scarcity of supplies and a lack of enforcement of legislation.

### Limitations

One of the limitations of this study is that results cannot be generalized and further studies with probability sampling methods need to be done.

## CONCLUSION

The present study highlighted a very low poor quality of life among sanitary workers. The majority of them are low paid workers, contract or daily wage employees. Their earnings are insufficient to meet their basic fundamental needs. They live in slums without any civic facilities and their working conditions are deplorable. They sweep the streets and gather trash without the use of gloves, masks or gowns. They go inside the drain without any proper clothing to open the clogged drains of the street and roadside. Their entire body, up to the neck, is submerged in sewage water. They are also not given any safety equipment.

## RECOMMENDATIONS

These workers are the backbone of civic life; without them, the city would be filthy and uninhabitable. Municipal authorities should provide good housing for them and their families, raise their wages and create a comprehensive service framework for these professional workers, as is done in other professions.

### Authors Contribution:

**SIAF:** Did data collection and manuscript writing.

**NM:** Conceived, study design, statistical analysis & editing of manuscript. He is also responsible for the integrity and accuracy of the study.

**SMM:** Did data analysis, interpretation, editing and final proof reading.
